# A simple strategy to effectively produce d-lactate in crude glycerol-utilizing *Escherichia coli*

**DOI:** 10.1186/s13068-019-1615-4

**Published:** 2019-11-20

**Authors:** Yao-De Wang, Jin-Yi Liao, Chung-Jen Chiang, Yun-Peng Chao

**Affiliations:** 1 Department of Chemical Engineering, Feng Chia University, 100 Wenhwa Road, Taichung, 40724 Taiwan; 2 Department of Medical Laboratory Science and Biotechnology, China Medical University, No. 91, Hsueh-Shih Road, Taichung, 40402 Taiwan; 3 Department of Medical Research, China Medical University Hospital, Taichung, 40447 Taiwan; 4 Department of Health and Nutrition Biotechnology, Asia University, Taichung, 41354 Taiwan

**Keywords:** Batch fermentation, Metabolic engineering, Crude glycerol, d-Lactate

## Abstract

**Background:**

Fed-batch fermentation has been conventionally implemented for the production of lactic acid with a high titer and high productivity. However, its operation needs a complicated control which increases the production cost.

**Results:**

This issue was addressed by simplifying the production scheme. *Escherichia coli* was manipulated for its glycerol dissimilation and d-lactate synthesis pathways and then subjected to adaptive evolution under high crude glycerol. Batch fermentation in the two-stage mode was performed by controlling the dissolved oxygen (DO), and the evolved strain deprived of *poxB* enabled production of 100 g/L d-lactate with productivity of 1.85 g/L/h. To increase productivity, the producer strain was further evolved to improve its growth rate on crude glycerol. The fermentation was performed to undergo the aerobic growth with low substrate, followed by the anaerobic production with high substrate. Moreover, the intracellular redox of the strain was balanced by fulfillment of the anaerobic respiratory chain with nitrate reduction. Without controlling the DO, the microbial fermentation resulted in the homofermentative production of d-lactate (ca. 0.97 g/g) with a titer of 115 g/L and productivity of 3.29 g/L/h.

**Conclusions:**

The proposed fermentation strategy achieves the highest yield based on crude glycerol and a comparable titer and productivity as compared to the approach by fed-batch fermentation. It holds a promise to sustain the continued development of the crude glycerol-based biorefinery.

## Background

The concern over environmental pollutions caused by petrochemicals-derived materials has urged a pressing need for biopolymers. Recognized as an eco-friendly material, polylactide (PLA) made from d- or l-lactic acid is featured with biocompatibility and processability [1]. PLA naturally hydrolyzes to the non-toxic constituent of α-hydroxy acid, which renders it appealing for biomedical applications [2]. Moreover, PLA can be processed in a mold due to its thermoplastic nature. Products of molded PLA are proved to be non-hazardous by the United State Food and Drug Administration and applicable for food packaging [3]. In particular, the stereocomplex PLA composed of poly d- and l-lactic acid displays a superior performance in terms of thermal stability and mechanical strength [4]. It has a potential application in the electronic sector and the automobile industry [5].

In addition to the synthesis of PLA, lactic acids of enantiomerical purity are required for the application in the textile, food, pharmaceuticals, and chemicals industries [6]. The chemical synthesis of lactic acid lies in the use of petrochemical resources and produces a racemic mixture containing d- and l-lactic acid. In contrast, the fermentation process implementing a producer strain with the desired trait enables the production of an optically pure lactic acid. The demand for l-lactic acid in the food industry has been historically fulfilled by performing the fermentation process with lactic acid bacteria (LAB) [3]. LAB are a diverse group of natural producer and capable of producing lactic acid with a high yield and high productivity. The LAB-based fermentation is conducted mainly with the glucose-rich feedstock [7].

The current price of PLA is not competitive with that of the petroleum-based plastics. To lower the production cost, the producer strain has to grow on a cost-effective medium and synthesize lactic acids with a titer > 100 g/L, a conversion yield close to the theoretical value, an optical purity > 99%, and high productivity [8]. The efficiency of a lactic acid-based production scheme closely links to the producer strain, the culture substrate, and the operational mode [6]. The growth of LAB generally requires a supplement of complex nutrients. Therefore, many studies have investigated a potential of surrogate bacteria involving *Bacillus* sp. [9], *Escherichia coli* [10], and *Corynebacterium glutamicum* [11]. Reported results for these strains receiving the genetic modification are generally encouraging. The lactic acid fermentation has been commonly conducted with glucose. However, the replacement of glucose with a renewable substrate reduces the production cost. Fed-batch fermentation circumvents the problem of substrate inhibition and enhances the production titer. The feeding strategy by maintaining glucose at a certain level was illustrated as an effective method for the production of lactic acids [12]. Nevertheless, it requires more labors and equipments to operate.

Crude glycerol appears to be a renewable resource which attracts the industrial attentions [13]. It is a by-product in the waste stream resulting from the production process of biodiesel. Social problems associated with the intensive use of fossil fuels have increased a demand for biodiesel. The refinement of crude glycerol for the production of value-added chemicals provides an appealing way to fulfill circular economy in the biodiesel industry [14]. In view of immaturity of the industrial fermentation process for d-lactic acid, we previously worked on the crude glycerol-based production of d-lactic acid in *E. coli*. The producer strain was obtained by manipulating its metabolic pathways leading to d-lactic acid. By fed-batch fermentation, the engineered strain enabled high production of d-lactic acid (> 99% optical purity) [15]. In this study, our continued effort was made to simplify the operational mode. A simple strategy based on batch fermentation was developed for the effective production of d-lactate from crude glycerol.

## Results and discussion

### Development of producer strain

**Fig. 1:**
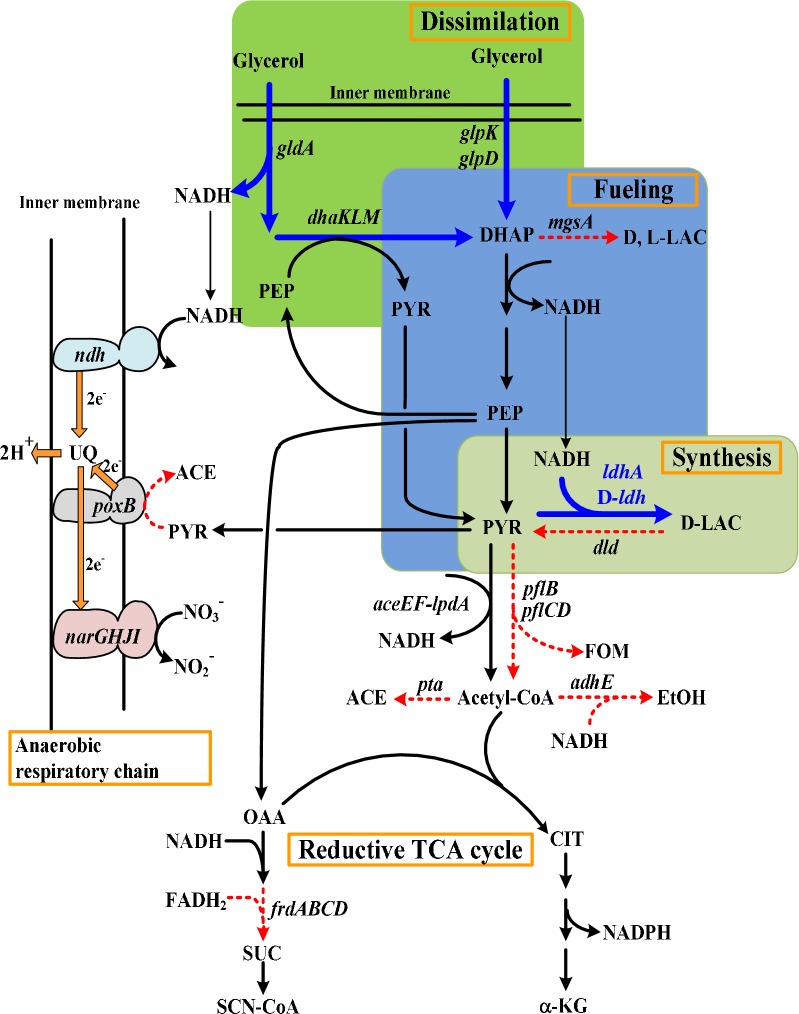
Metabolic pathways of interest leading to the synthesis of d-lactate from glycerol. The enhanced and blocked pathways were highlighted in blue (solid line) and red (dotted line), respectively. Genes and key metabolites involved in metabolic pathways include as follows: *aceE*-*lpd*, pyruvate dehydrogenase; *adhE*, aldehyde–alcohol dehydrogenase; *dld*, *FAD*-*linked* d-lactate dehydrogenase; *dhaKLM*, dihydroxyacetone kinase operon; *frdABCD*, fumarate reductase operon; *ldhA*, NAD-linked d-lactate dehydrogenase; *D*-*ldh*, *L. helveticus* d-lactate dehydrogenase; *mgsA*, methylglyoxal synthase; *narGHJI*, nitrate reductase operon*; ndh,* NADH dehydrogenase; *poxB*, pyruvate oxidase; *gldA*, glycerol dehydrogenase; *glpD*, glycerol 3-phosphate dehydrogenase; *glpK*, glycerol kinase; *pflB*, pyruvate-formate lyase; *pflDC*, putative pyruvate-formate lyase; *pta*, phosphate acetyltransferase; *ACE* acetate, *CIT* citrate, *DHAP* dihydroxyacetone phosphate, *EtOH* ethanol, *FOM* formate, *α-KG* α-ketoglutarate, *D-LAC* d-lactate, *L-LAC* l-lactate, *OAA* oxaloacetate, *PEP* phosphoenolpyruvate, *PYR* pyruvate, *SUC* succinate, *SCN-CoA* succinyl-CoA, *UQ* ubiquinone

In our previous work, fed-batch fermentation of strain BLac-2106 was illustrated to effectively produce d-lactate using crude glycerol in terms of a high titer and high productivity [15]. In essence, this strain was deficient in *adhE*, *frdA*, *pflB*, and *pta* to curtail the carbon waste for the synthesis of byproducts including ethanol, formate, succinate, and acetate (Fig. 1). To enhance the synthesis of d-lactate, the strain was equipped with a genomic copy of the λP_L_ promoter (PλP_L_)-driven d-*ldh* from *Lactobacillus helveticus* at *pflCD*. The strain was also deprived of *dld* and *mgsA*, which renders it unable to metabolize the enantiomerical purity of d-lactate. Although the fermentation result is encouraging, the fed-batch mode is complicated and unfriendly to operate. Therefore, this study was aimed to simplify the production scheme. As indicated in Fig. 1, the synthetic route leading to d-lactate from glycerol generally contains 3 module pathways participating in glycerol dissimilation, the supply of pyruvate precursor and energy, and the d-lactate synthesis. The dissimilation and synthesis pathways were reasoned to be limited. Therefore, strain BLac-2106 was further modified by genomic fusion of PλP_L_ with endogenous *gldA*, *dhaKLM*, and *ldhA*. The latter encodes lactate dehydrogenase (LDH) responsible for the conversion of pyruvate into d-lactate [16]. The catalytic function of GldA and DhaKLM mediates the fermentative dissimilation of glycerol [17]. The modified strain displayed a 40% increase in the LDH. The expression level of *gldA* and of *dhaKLM* in the strain was elevated by 45% and 80%, respectively. The implementation of batch fermentation to achieve a high titer is challenging because of substrate inhibition. The modified strain was, thus, subjected to the adaptive evolution, and one resulting strain (designated EcoB-140) was scored for further characterization (Additional file 1: Figure S1).

### Production of d-lactate by batch fermentation

**Fig. 2:**
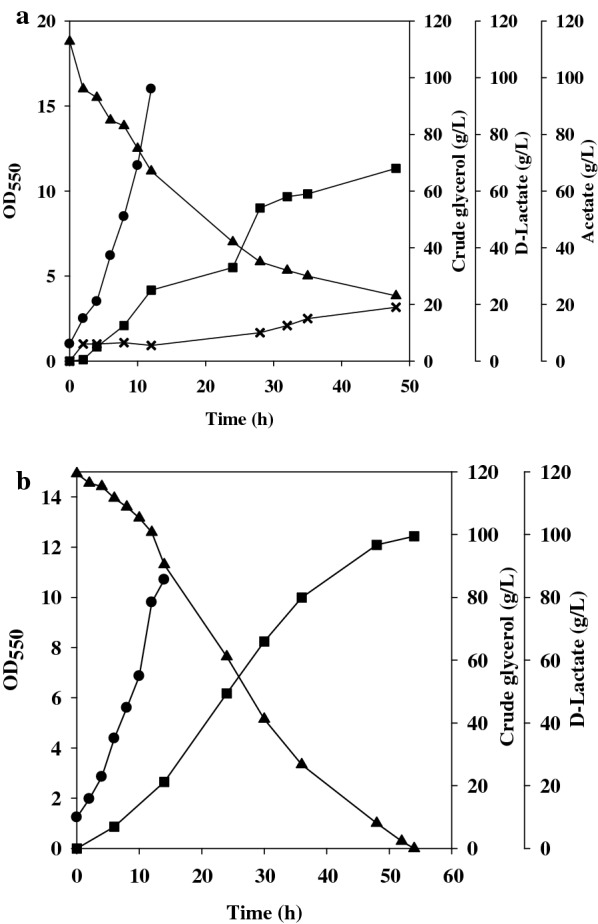
Production of d-lactate by batch fermentation. The engineered strain was cultured in a bench fermenter containing crude glycerol. The batch fermentation was under the control of the DO to proceed the aerobic growth phase and the fermentative production phase. The fermentation was initiated with the cell density at OD_550_ of 1. The absorbance measurement for the cell density in the production phase was interfered with crude glycerol and Ca(OH)_2_ and, therefore, not reported. The experiment was duplicated. The typical fermentation profiles were reported for strain EcoB-140 (**a**) and strain EcoB-140B (**b**). Cell density (black circle); crude glycerol (black up-pointing triangle); d-lactate (black square); acetate (X)

Batch fermentation in the two-stage mode was conducted with strain EcoB-140 using crude glycerol (110 g/L). This was first performed with high aeration for rapid accumulation of cell biomass (i.e. the growth phase). Meanwhile, yeast extract (10 g/L) was supplemented to eliminate the lag growth of the strain. After the termination of bacterial growth at 12 h of the fermentation, the production phase was initiated simply by lowering the dissolved oxygen (DO) tension to 5% of the saturated level. At the end of the fermentation, the strain produced d-lactate of 70 g/L at the expense of 90 g/L crude glycerol (Fig. 2a). A large portion of d-lactate appeared in the production phase.

It was unexpected to detect the production of acetate for the strain grown on a high concentration of glycerol (Fig. 2a). Our previous study illustrated the implementation of fed-batch fermentation for the production of d-lactate with negligible byproducts [15]. This was carried out by a stepwise feeding of glycerol to keep its level below 10 g/L during the fermentation process. Overall, acetate overflow likely correlates with the glycerol level. Pyruvate oxidase encoded by *poxB* in *E. coli* catalyzes the oxidative decarboxylation of pyruvate to acetate, and its expression level is induced in the presence of glycerol and in response to osmotic stress as well [18]. Accordingly, a high expression level of *poxB* could be realized in the strain exposed to a high concentration of glycerol. The absence of acetate in the glycerol-grown *E. coli* was due to the reutilization of acetate mediated by *acs*-encoded acetyl-CoA synthetase [18]. Acetate overflow occurring in strain EcoB-140 is likely the result of the synthesis rate exceeding the utilization rate. Accordingly, strain EcoB-140 was deprived of *poxB* to give strain EcoB-140B. Batch fermentation of the resulting strain was carried out with crude glycerol (120 g/L) and yeast extract (10 g/L). As shown in Fig. 2b, strain EcoB-140B consumed around 30 g/L glycerol in the first 12 h of the fermentation (i.e., the growth phase). In the production phase, d-lactate started to largely accumulate and reached around 100 g/L at 53 h of the fermentation. Acetate was not detected at the end of the fermentation.

### Production of d-lactate by the two-dose fermentation

**Fig. 3:**
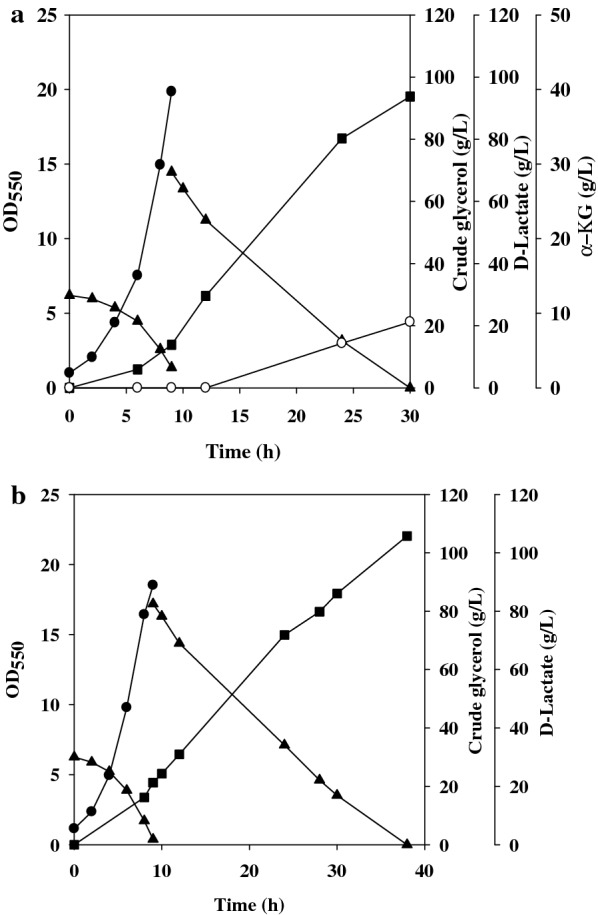
Production of d-lactate by the two-dose fermentation. The fermentation was conducted with the aerobic growth phase and the fermentative production phase. A high dose of crude glycerol was added to the culture broth at the end of the growth phase. The fermentation was initiated with the cell density at OD_550_ of 1. The absorbance measurement of the cell density in the production phase was interfered with crude glycerol and Ca(OH)_2_ and, therefore, not reported. The experiment was duplicated. The typical fermentation profiles were reported for the cases with the DO control (**a**) and without the DO control (**b**). Cell density (black circle); crude glycerol (black up-pointing triangle); d-lactate (black square); α-KG (white circle)

Crude glycerol drastically perturbs the physiological status of microbes [19]. It appears that the engineered strain is susceptible to the stress incurred by a high concentration of crude glycerol, which in turn retards the bacterial growth and decreases productivity. This issue may be addressed by the approach using a high cell density. Batch fermentation with two substrate doses (i.e., two-dose fermentation) was, thus, performed by growing the strain on a low dose of crude glycerol, followed by adding a high dose to the culture upon initiation of the production phase. The strain was first cultured with high aeration in the presence of crude glycerol (30 g/L) plus yeast extract (2 g/L). As shown in Fig. 3a, the strain almost consumed all glycerol at 9 h of the fermentation. Subsequently, crude glycerol of 70 g/L was added into the fermentation broth and the DO was controlled at around 5% of the saturated level. At 30 h of the fermentation, the strain consumed all glycerol and produced d-lactate of 92 g/L. The result indicates that this proposed strategy has a potential to improve the strain’s performance on crude glycerol in terms of productivity (from 1.85 to 3.1 g/L/h).

Unexpectedly, the fermentation ended with the associated production of α-ketoglutarate (α-KG) in the strain (Fig. 3a). The tricarboxylic acid (TCA) cycle proceeds to the reductive pathway consisting of two terminal nodes at succinyl-CoA and α-KG in fermenting *E. coli* (Fig. 1) [20]. The lack of *frdA* (encoding fumarate reductase) conserves oxaloacetate (OAA) in strain EcoB-140B, which in turn favors the catalytic conversion of available OAA with acetyl-CoA to citrate. Citrate is further oxidized to α-KG. It is recognized that the low oxygen tension negates the functional activity of the TCA cycle [21]. In the absence of oxygen, *E. coli* relies on either nitrate reductase or fumarate reductase to reduce nitrate or fumarate which serves as the terminal electron acceptor in the respiratory chain [22]. Accordingly, the production phase was conducted under the low oxygen tension. To further simplify the operation, the fermentation was carried out without controlling the DO. The strain was cultured on crude glycerol (30 g/L) plus yeast extract (2 g/L) for 9 h with a high agitation at 500 rpm in the growth phase. In the second phase, crude glycerol (80 g/L) and NH_4_NO_3_ (20 mM) were added to the culture broth and the fermentation proceeded to the production of d-lactate with a low agitation at 200 rpm. It was found that the DO fluctuated below 1% of the saturated level. The strain consumed all glycerol and produced 105 g/L d-lactate at 38 h of the fermentation (Fig. 3b). α-KG was not detected at the end of the fermentation. Nitrate reductase is expressed in response to nitrate and fulfills its role in the respiratory chain of fermenting bacteria. As a result, it provides an electron sink to balance the intracellular redox level (Fig. 1). Note that NADH dehydrogenase encoded by *ndh* primarily functions in the nitrate respiration [23].

### Effective production of d-lactate

**Fig. 4:**
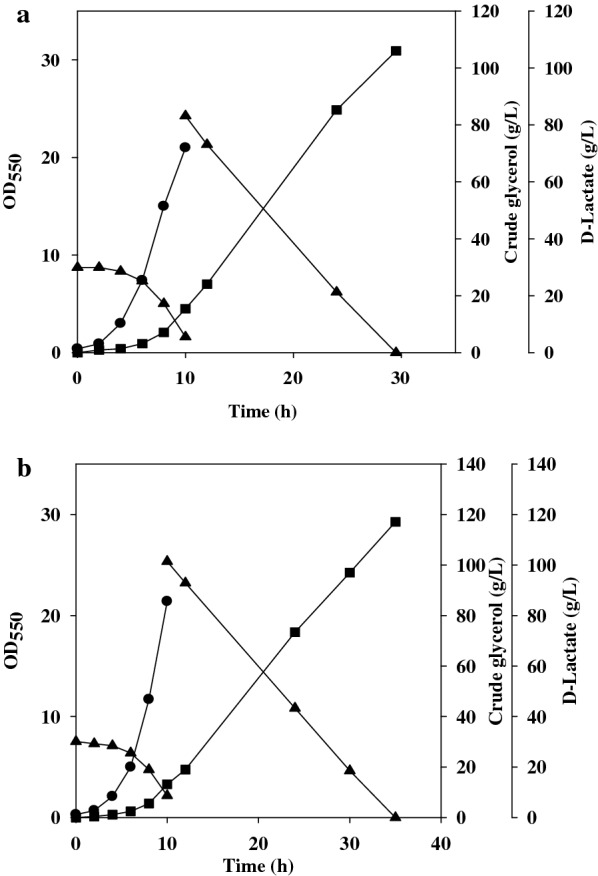
Effective production of d-lactate by the two-dose fermentation. Refer to Fig. 3 for the fermentation process. The fermentation was initiated with the cell density at OD_550_ of 0.2. The absorbance measurement of the cell density in the production phase was interfered with crude glycerol and Ca(OH)_2_ and, therefore, not reported. The experiment was duplicated. The typical fermentation profiles were reported for strain EcoB-170 (**a**) grown on 2 g/L yeast extract and fed with 80 g/L crude glycerol and (**b**) grown on 1 g/L yeast extract and fed with 90 g/L crude glycerol. Cell density (black circle); crude glycerol (black up-pointing triangle); d-lactate (black square)

The *poxB*-deficient strain indeed displayed a slower growth rate, in agreement with the growth defect of the mutant strain on glucose [24]. The PoxB-mediated reaction is coupled to the respiratory chain via ubiquinone and generates the proton motive force [25]. The strain without PoxB is likely in the shortage of energy. To improve its growth, strain EcoB-140B was evolved in the presence of 30 g/L crude glycerol without yeast extract. Finally, one strain (designated EcoB-170) displaying better growth was scored for investigation. The two-dose fermentation of strain EcoB-170 was carried out as described earlier except that the initial cell density (i.e., 0.2 at OD_550_) was reduced to one fifty of that used in Fig. 3b. As a result, the strain solely produced d-lactate with a titer of 105 g/L at 30 h of the fermentation (Fig. 4a). The performance of strain EcoB-170 was further investigated for its growth on a lower level of yeast extract (i.e., 1 g/L). The production phase was initiated with a higher substrate dose (i.e., 90 g/L). It consequently resulted in d-lactate of 115 g/L at 35 h of the fermentation (Fig. 4b).

The fermentative production of d-lactate has been mostly conducted with glucose. There are few studies reporting the use of crude glycerol. In one report, *E. coli* strain B0013-070 was constructed with overexpression of *ldhA* and the deletion of *ackA*-*pta*, *adhE*, *dld*, *frdA*, *pflB, poxB,* and *pps* [26]. The fermentation was conducted with the aerobic phase at 34 °C, followed by the microaerobic phase at 40 °C. The DO was controlled below 1% of the saturated level and the glycerol level was maintained above 10 g/L during the microaerobic phase. As a result, the strain produced d-lactate of 100 g/L with the conversion yield and productivity reaching 0.75 g/g and 2.78 g/L/h, respectively (Table 1). Our previous work reported the development of strain BLac-2106 by genetic manipulation of the central metabolism, particularly the glycerol catabolism [15]. The production phase was proceeded to maintain the DO at 1% of the saturated level by adjusting the feeding dose of glycerol after the termination of the growth phase. The implementation of fed-batch fermentation enabled the engineered strain to produce d-lactate with a titer of 105 g/L, a yield of 0.75 g/g, and productivity of 2.78 g/L/h. Others with the emphasis on pure glycerol have been also reported. One study was conducted with the *thiE*-null strain B0013-080A which was unable to synthesize thiamine monophosphate [27]. The growth of strain B0013-080A was controlled by the availability of supplemented thiamine, and d-lactate was produced for the non-growing strain upon the depletion of thiamine. Fed-batch fermentation was carried out by dosing 4 batches of glycerol, and the strain produced d-lactate with a titer of 120 g/L, a yield of 0.87 g/g, and productivity of 4.11 g/L/h. Another work reported the isolation of *E. coli* strain AC-521 from soil samples. By keeping glycerol between 20 and 30 g/L, the fermentation of the strain in a fed-batch mode led to the production of lactic acid of 86 g/L at 40 °C [28]. The result accounts for a yield of 0.88 g/g and productivity of 0.97 g/L/h. In addition, *Klebsiella pneumoniae* was deprived of *dhaT* and *yqhD* to block the synthetic pathway of 1,3-propanediol [29]. Glycerol was continuously fed at a constant rate in the microaerobic phase. By fed-batch fermentation, the strain with the overexpression of *ldhA* enabled production of 142 g/L d-lactate with a yield of 0.82 g/g and productivity of 2.96 g/L/h. Nevertheless, the fermentation with *K. pneumoniae* requires the supplement of peptone and beef extract.

**Table 1:** d-Lactate production by microbes utilizing glycerol

Strain	Operation	Substrate	Titer (g/L)	Yield (g/g)	Productivity (g/L/h)	References
*E. coli*						
EcoB-140B	Batch	Crude glycerol	100^b^	0.95^c^	1.85^d^	This study
EcoB-170	Two-dose^a^	Crude glycerol	115^b^	0.97^c^	3.29^d^	This study
BLac-2106	Fed-batch	Crude glycerol	105	0.87	2.63	[15]
AC-521	Fed-batch	Pure glycerol	86	0.88	0.97	[28]
B0013-070	Fed-batch	Crude glycerol	100	0.75	2.78	[26]
B0013-080A	Fed-batch	Crude glycerol	120	0.87	4.11	[27]
*Klebsiella pneumoniae*						
ATCC25955	Fed-batch	Pure glycerol	142	0.82	2.96	[28]

^a^The batch fermentation was conducted with the administration of two substrate doses

^b^The measured titer was the apparent concentration of d-lactate in the fermentation broth

^c^The yield based on crude glycerol was calculated by multiplication of the measured titer with the culture volume plus the added volume of the neutralizing solution

^d^Productivity was calculated by dividing the measured titer with the whole fermentation time (time zero to the end of the fermentation)

The complication of controlling the substrate level in a bioreactor makes fed-batch fermentation practically ineffective. Moreover, the operation usually needs sophisticated devices which increase the equipment investment. Batch fermentation apparently simplifies the production process without these drawbacks. However, the problem of substrate inhibition presents to be challenging. The brewing industry has implemented the very high gravity fermentation (VGHF) technology to remain profitable [30]. Ethanolic yeast is afflicted with the disorder in membrane fluidity and glucose metabolism under the hypertonic environment originating from VGHF [31]. The cellular utilization of glucose is retarded by the decreased expression of certain glycolytic enzymes [32]. A defect in membrane fluidity handicaps the solute transport systems of cells [33]. In contrast, a high level of crude glycerol induces the inhibitory effect on *E. coli* and the underlying mechanism may vary with the various compositions of crude glycerol. The producer strain was, thus, evolved in the hypertonic solution of crude glycerol. The supplement of yeast extract helped the cell growth in batch fermentation, whereas acetate overflow occurred for the evolved strain (Fig. 2a). This issue was addressed by deletion of *poxB* in the strain. The resulting strain enabled production of 100 g/L d-lactate with a yield of 0.95 g/g and productivity of 1.85 g/L/h (Table 1). To improve productivity, the strain was further evolved (i.e., strain EcoB-170) and was then employed for the two-dose fermentation. This process was first conducted with a light dose in the bioreactor to favor the aerobic growth. In the second stage, a heavy dose was added to speed up the anaerobic catabolism of glycerol. Note that this approach leads to a drastic reduction in yeast extract and initial cell density as applied for the microbial fermentation, which lowers the production cost. Consequently, strain EcoB-170 produced the optically pure d-lactate (> 99%) with a titer of 115 g/L, a yield of 0.97 g/g, and productivity of 3.29 g/L/h (Table 1). As previously illustrated, the bacterial growth was inhibited by peroxides (at μM) occurring in crude glycerol [34]. Tert-butyl hydroperoxide (TBHP) was identified as the major compound contributing to the cytotoxicity. The effect of TBHP on strain EcoB-170 was then investigated by culturing the strain with 20 g/L pure glycerol plus TBHP ranging 60–90 μM. Consequently, the cell growth remained unaffected. Another study reported that the lactic acid–iron complex mediated the generation of the reactive hydroxyl radical (HO^.^) from peroxides [35]. Taken together, strain EcoB-170 is likely evolved to tolerate the induced oxidative stress. Nevertheless, the impurities of crude glycerol vary with various feedstock origins. There is need for further investigation of the detailed mechanism which underlines the resistance of the strain to crude glycerol.

## Conclusions

In conclusion, this study proposed a simple fermentation technology. This fermentation strategy can be easily operated without the DO control. The result of this approach achieves the highest yield based on glycerol and a comparable titer and productivity as compared to those by fed-batch fermentation (Table 1). This proposed technology holds a promise to sustain the continued development of the crude glycerol-based biorefinery.

## Methods

### Strain construction and adaptive evolution

To enhance the expression level, endogenous *gldA, dhaKLM,* or *ldhA* was fused with PλP_L_. This construction work essentially followed the reported protocol. In brief, the LE*-*gen*-RE*-PλP_L_ cassette flanked by two homologous extensions was amplified from plasmid pPL-Gn by PCR with Gld1–Gld2 or Dha1–Dha2 primers [15]. By the act of λ Red, the genomic locus was targeted by recombination with the electroporated PCR DNA in the host strain. The integrant strain was scored after removal of the inserted marker (i.e., *gen*) using Cre [36]. To delete *poxB*, the truncated gene was amplified from strain JW2121-1 (△*poxB*::FRT-*kan*-FRT) by PCR with the primers (cgctgaaggttacgtactgg and tagtcctgcagagcattaacg). The λ Red-mediated recombination resulted in the integration of the PCR DNA into the genome of the strain, and the *kan* marker flanked by FRT was removed using Flp [37].

Crude glycerol was provided by Great Green Technology (Changhua, Taiwan). Adaptive evolution of the strain was carried out by a serial of cell subculture with the increasing level of crude glycerol. In general, the to-be-evolved strain was cultured in the Erlenmeyer flask (125 mL) containing NBS medium (20 mL) plus the indicated amount of crude glycerol with or without yeast extract. The bacterial culture was incubated at 37 °C with vigorous shaking for 24 h, and the cell density was measured turbidimetrically at 550 nm (OD_550_). Cell subculture was prepared by transferring the adapted culture to a flask containing the fresh medium. The cell subculture was repeated as needed.

### Bacterial fermentation

Batch fermentation was performed as follows. The overnight culture was routinely prepared with a flask containing Luria–Bertani (LB) medium [38]. The experiment was conducted by inoculating the overnight culture into flasks containing 100 mL NBS medium [15] with 20 g/L crude glycerol. The seeding culture was incubated at 37 °C with vigorous shaking for 12 h. Bacteria were harvested by centrifugation and inoculated into a bioreactor (New Brunswick Bioflow 110) containing 1-L NBS medium plus crude glycerol and yeast extract as indicated. The initial cell density was maintained at OD_550_ of 1 or 0.2. The operation condition was set up as follows unless stated otherwise. In the growth phase, the DO of the fermentation broth was maintained at 30% of the saturated level by purging air at 1 vvm and the automatic control of the agitation rate. In the production phase, the DO was controlled at 3% of the saturated level throughout the fermentation. Alternatively, crude glycerol (80–90 g/L) was added into the culture broth and the fermentation was performed without controlling the DO. The fermentation was conducted at 37 °C and the broth pH was maintained at 7 using 20% (w/v) Ca(OH)_2_.

### Enzyme assay

The enzyme activity of lactate dehydrogenase was determined based on the previous report [15]. The cell-free extract was prepared and added to the reaction solution consisting of 1-mM pyruvate, 0.053-mM NADH, and 100-mM sodium phosphate buffer (pH 7.0). The reaction was carried out at 25 °C and the absorbance at 340 nm was measured along the time course. The enzyme activity was calculated according to the reduction in the NADH level in μmol per min.

Following the reported protocol [15], the expression level of *gldA* and *dhaK* was quantified by calculation of the individual mRNA level normalized to that of *ihfB*. In brief, total RNAs of the strain were isolated by Ambion^®^ RiboPure (Life technology, USA) and used to synthesize the corresponding cDNAs with High Capacity cDNA Reverse Transcription kit (Life technology), the Power SYBR^®^ Green PCR Master Mix and Power SYBR^®^ Green RT-PCR Reagent kit. The real-time PCR analysis was carried out using the Applied Biosystems^®^ StepOne Real-Time PCR System (Life technology).

### Fermentation analysis

High-Performance Liquid Chromatography AS3000 (Thermo Scientific, USA) was used for the fermentation analysis. Glycerol was determined with the ICSep ICE-ION-300 column (Transgenomic, USA) and a RI detector. Organic acids were measured with the ICSep ICE-ORH-801 column (Transgenomic, USA) and a UV detector set at 210 nm. The mobile phase consisting of 0.0085 N H_2_SO_4_ was used for the analysis. Analysis methods essentially followed the previous report [15].

## Supplementary information

**Additional file 1: Figure S1.** The time course of the *E. coli* strain undergoing adaptive evolution.

## Data Availability

Not applicable.

## Abbreviations

α-KG: α-ketoglutarate
DO: dissolved oxygen
LAB: lactic acid bacteria
LDH: lactate dehydrogenase
PλP_L_: λP_L_ promoter
LB: Luria–Bertani
OAA: oxaloacetate
PLA: polylactide
TCA: tricarboxylic acid
TBHP: tert-butyl hydroperoxide
VGHF: very high gravity fermentation
