# Supplementary material for: A simple strategy to effectively produce d-lactate in crude glycerol-utilizing Escherichia coli
Source: Biotechnol Biofuels. 2019 Nov 20;12:273. doi: 10.1186/s13068-019-1615-4 (PMC6864932; doi:10.1186/s13068-019-1615-4)
Supplement: Supplementary file 1 — Additional file 1: Figure S1. The time course of the E. coli strain undergoing adaptive evolution. [file 13068_2019_1615_MOESM1_ESM.pdf]

Additional file 1
for
A simple strategy to effectively produce D-lactate in crude glycerol-utilizing *Escherichia coli*

Yao-De Wang,[1] Jin-Yi Liao,[1] Chung-Jen Chiang,[2,*] and Yun-Peng Chao,[1,3,4*]

[1]Department of Chemical Engineering, Feng Chia University, 100 Wenhwa Road, Taichung, Taiwan 40724

[2] Department of Medical Laboratory Science and Biotechnology, China Medical University, No. 91, Hsueh-Shih Road, Taichung, Taiwan 40402

[3]Department of Medical Research, China Medical University Hospital, Taichung 40447, Taiwan

[4]Department of Health and Nutrition Biotechnology, Asia University, Taichung 41354, Taiwan

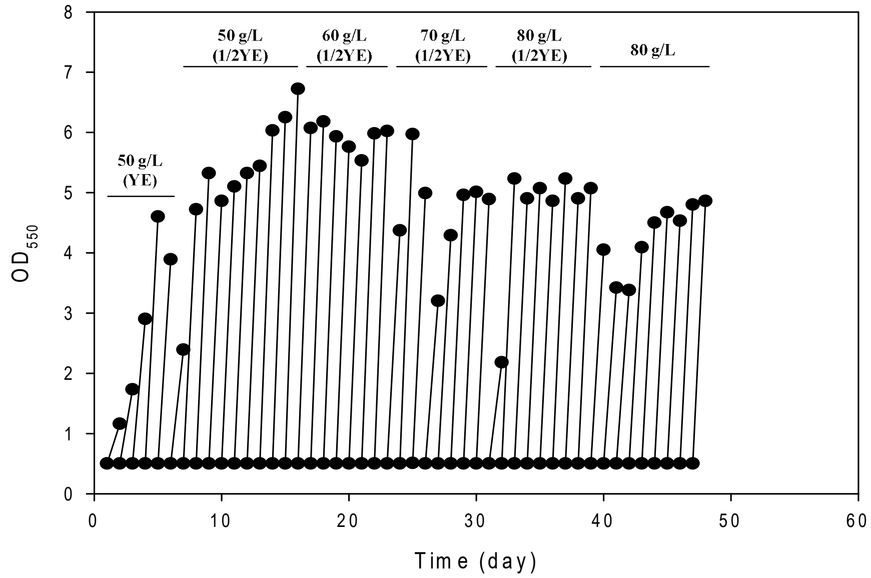

Figure S1 The time course of the *E. coli* strain undergoing adaptive evolution. The strain was first cultured in a shake flask containing crude glycerol (50 g/L) plus yeast extract (YE) of 10 g/L. The serial subculture was performed every 24 h. During the evolution course, YE was reduced by half (1/2 YE) and crude glycerol was gradually increased as indicated. Finally, the strain was transferred to the medium containing 80 g/L crude glycerol without YE.
